# Potential application of sucrose acetate isobutyrate, and glyceryl monooleate for nanonization and bioavailability enhancement of rivaroxaban tablets

**DOI:** 10.1016/j.pscia.2023.100015

**Published:** 2023-10-14

**Authors:** Adam A. Al-Shoubki, Mahmoud H. Teaima, Rehab Abdelmonem, Mohamed A. El-Nabarawi, Sammar Fathy Elhabal

**Affiliations:** a Department of Pharmaceutics and Industrial Pharmacy, Faculty of Pharmacy, University of Derna, Derna, Libya; b Department of Pharmaceutics and Industrial Pharmacy, Faculty of Pharmacy, Cairo University, Cairo, 11562, Egypt; c Department of industrial Pharmacy, College of Pharmaceutical Sciences and Drug Manufacturing, Misr University for Science and Technology (MuST), 6th of October City, Giza, 12566, Egypt; d Department of Pharmaceutics and Industrial Pharmacy, Faculty of Pharmacy, Modern University for Technology and Information (MTI), Mokattam, Cairo, 11571, Egypt

**Keywords:** Nanonization, Rivaroxaban, Sucrose acetate isobutyrate, Glyceryl monooleate, Bioavailability enhancement

## Abstract

This study aimed to investigate the use of Sucrose acetate isobutyrate (SAIB) and Glyceryl monooleate (GMO) as co-formers for creating Cubosomes and SAIB-based nanodispersions of Rivaroxaban (RXB). The process utilized a modified melt dispersion technique with varying polymer: drug ratios (0.5:1, 0.75:1, and 1:1) and a fixed polymer: poloxamer 407 ratio (0.1:1). Particle size (PS), polydispersity index (PDI), zeta potential (ZP), and entrapment efficiency (EE) were measured to determine the optimal formulas. The best-lyophilized formulas were then analyzed using Fourier transform infrared spectroscopy (FT-IR), powder X-ray diffraction (PXRD), differential scanning calorimetry (DSC), dissolution testing, and Pharmacokinetic (PK) studies. The results revealed significant correlations between polymer concentrations and various variables in cubosomal and SAIB-based nanodispersions. An increase in GMO concentration led to a decrease in PS, PDI, and ZP but an increase in EE and yield. Maintaining optimal GMO concentration is crucial for consistent nanoparticle formulations. In contrast, increasing SAIB concentration led to a decrease in PS and PDI but an increase in EE and yield. The drug release rates of different preparations were measured during the dissolution test. The best-lyophilized cubosome (L4) and the best-lyophilized SAIB-based nanodispersions (L8) showed significantly improved drug release compared to XARELTO®. L4 displayed the best dissolution rate, and L8 also had a reasonable rate. A PK study demonstrated that L4 and L8 had significantly better bioavailability than XARELTO®, possibly due to their improved solubility. This study suggests that SAIB and GMO can significantly enhance the solubility and bioavailability of RXB in nano preparations, leading to more efficient drug delivery. This new approach can also reduce the required dosage for the desired therapeutic effect. However, further research is needed to fully understand these polymers' potential benefits and limitations.

## 1 Introduction

Rivaroxaban (RXB), which is the active pharmaceutical ingredient (API), was approved in July 2011 for treating deep vein thrombosis and pulmonary embolism [1,2]. Recently, RXB has been used more frequently to prevent thrombosis in non-valvular atrial fibrillation (NVAF) patients, obese patients, and hospitalized patients with Coronavirus disease (COVID-19) [3,4]. Unfortunately, RXB's low water solubility leads to low bioavailability, a common issue for 40% of poorly soluble drugs on the market [5,6]. RXB is considered a BCS class II drug with a 60% bioavailability rate for a 20 ​mg dose under fasting conditions [7,8]. However, the bioavailability of RXB is dose-dependent, with a 90% bioavailability rate for a 10 ​mg dose and a 100% bioavailability rate for a 5 ​mg dose [8]. Additionally, food can affect the bioavailability of RXB at a 15–20 ​mg dose [9]. The low solubility of RXB makes it challenging to formulate and administer, leading to inconsistent absorption and reduced efficacy that can impact its clinical outcomes. Therefore, enhancing the solubility of RXB is crucial for improving its therapeutic efficacy and clinical utility.

**Fig. 1:**
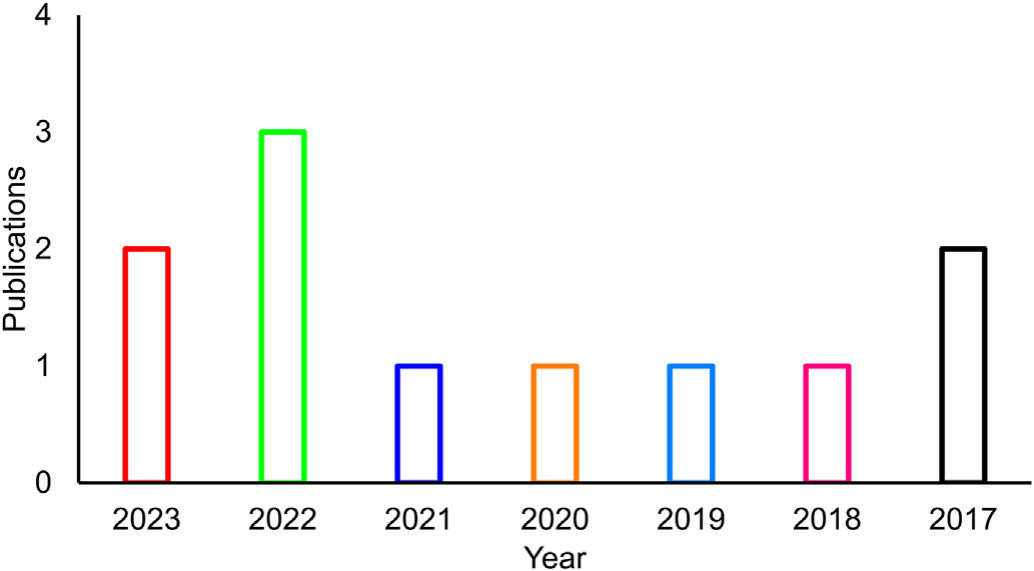
Cumulative number of publications on enhanced oral bioavailability of RXB. The number of publications was determined by searching the PubMed database (https://pubmed.ncbi.nlm.nih.gov/) on 12 July 2023.

However, there are very limited studies on enhancing the bioavailability of the RXB (as shown in Fig. 1), including Microspheres [10], Liposomes [11], Self-Nanoemulsifying Drug Delivery System [12], Solid Lipid Nanoparticles (SLNs) [13], Cocrystals [8], Sustained release [14], Solid dispersion [15]. Unfortunately, each has advantages and disadvantages that require careful consideration. As a result, further research and development are necessary to optimize the solubility of RXB and maximize its therapeutic potential.

Sucrose acetate isobutyrate (SAIB) has become increasingly popular in the pharmaceutical industry due to its exceptional capacity to enhance drug delivery and effectiveness via the production of drug nanoparticles. The FDA has approved it for food and pharmaceutical usage, and it is entirely non-toxic and biodegradable. SAIB is adept at controlling particle size and shape, stabilizing nanoparticles, and extending their shelf life. Given its versatility and safety, SAIB holds great promise as a vital component in future pharmaceutical applications. [16,17]. A recent study has shown that SAIB exhibits promising potential in preparing oral nanovesicles to improve drug solubility [18]. Glyceryl monooleate (GMO) is a well-known surfactant due to its biocompatibility, biodegradability, and non-toxic nature [19,20]. Researchers have demonstrated the successful preparation of cubic-phase lipid nanoparticles using GMO since the 1990s [[21], [22], [23], [24]]. These nanoparticles, specifically the GMO/poloxamer 407 cubic nanoparticles, have been researched as a potential oral drug delivery vehicle to increase the bioavailability of poorly soluble drugs [[24], [25], [26]]. Additionally, both SAIB and GMO display mucoadhesive behavior which can potentially enhance drug delivery [27,28]. Our study focuses on developing nano-vesicular systems and cubosomes for RXB, utilizing SAIB and GMO as co-formers, and examining their pharmaceutical properties. While GMO has been studied extensively for this purpose, SAIB is less widely discussed. We utilized a modified melt dispersion technique to create cubosomes and nano-vesicular systems.

## 2 Materials and methods

### 2.1 Materials

RXB, Batch no. RB1907004 (Megafine Pharma (P) Ltd; Maharashtra, India); was utilized in this study as an example of a new low water-soluble drug. 20 ​mg Xarelto® tablets (Bayer, Leverkusen, Germany) were bought. SAIB, Batch no. K-190064 (Sigma-Aldrich, Missouri, USA), and GMO, Lot no. 080737001 (Supelco, Pennsylvania, USA) were evaluated as possible nanonization-inducing excipients. Poloxamer 407 (P407), Lot no. GNC22321B (Sigma Pharmaceutical Industries, Quesna, Egypt) using as a stabilizer and solubility enhancer of hydrophobic molecules in polar media. Mannitol (MAN), Batch no. 1812072 (Shijiazhuang Huaxu Pharmaceutical Co., Ltd, Shijiazhuang, China) is used as a binder, bulking agent, and to protect the formula from degradation upon lyophilization. Acetonitrile (Oxford, Bombay, India), disodium mono-hydrogen phosphate, and potassium dihydrogen orthophosphate (El-Gomhoria Company for Chemicals Cairo, Egypt). The other compounds were of analytical quality.

### 2.2 Methods

#### 2.2.1 Preparation of the cubosomal and SAIB-based nano-vesicular (SBN) dispersions

A modified melt dispersion technique was utilized to create cubosomal and SAIB-based nano-vesicular (SBN) dispersions. This involved liquefying GMO or SAIB at 70°C in a water bath and adding RXB and P407 (Table 1). After stirring to create a homogeneous mixture, it was slowly added to distilled water while stirring at 1000 ​rpm. The dispersion was then stirred until it reached room temperature and ultrasonicated for 15 ​min using a probe sonicator (Cole-Parmer, Alaska, USA). Finally, the preparations were stored in the refrigerator until analysis.

**Table 1:** Composition and physicochemical characterization of cubosomal and SAIB-based nanodispersions.

Formula	W/W ratio				PS (nm)	PDI	ZP (mv)	EE (%)	Yield (% w/w)
	Drug	GMO	SAIB	P407					
F1	1	0.5	–	0.30	139.4 (±9.45)	0.372 (±0.33)	−11.76 (±0.75)	68.53 (±3.46)	101.2 (±0.44)
F2	1	0.75	–	0.35	130.0 (±7.54)	0.273 (±0.46)	−17.96 (±1.30)	72.16 (±3.92)	99.67 (±0.29)
F3	1	1	–	0.40	111.7 (±6.00)	0.190 (±0.02)	−24.99 (±1.28)	80.36 (±6.81)	104.6 (±0.85)
F4	1	–	0.5	0.30	512.3 (±27.0)	0.737 (±0.04)	−3.58 (±0.46)	55.20 (±4.43)	100.0 (±0.25)
F5	1	–	0.75	0.35	252.5 (±3.93)	0.193 (±0.02)	−3.73 (±0.56)	54.93 (±4.52)	99.67 (±0.29)
F6	1	–	1	0.40	175.6 (±9.45)	0.254 (±0.05)	−4.03 (±0.67)	79.73 (±3.05)	101.5 (±0.36)

#### 2.2.2 HPLC analysis

With great precision and accuracy, the HPLC analysis of RXB was conducted using a methodology based on previous research by Gouveia et al., 2020 and Meng et al., 2022 with slight modifications [8,29]. The Knauer AZURA HPLC Plus System with a Diode array detector (product no: A46007, Berlin, Germany) was utilized to analyze the drug concentrations. The data was processed using the Knauer ClarityChrom® software package, version 8.5.0. The reversed-phase C18 column (LiChroCART®Purospher®Star C18 column; 55 ​mm ​× ​4 ​mm, 3 ​μm particle size) was maintained at a temperature of 30°C, and the separation of RXB was achieved within a few minutes. The mobile phase composition consisted of A:B 40:60 (v/v), where (A) water contains 0.1% formic acid and (B) acetonitrile. The flow rate was set at 1.0 ​ml/min, the wavelength was set at 249 ​nm, and the injection volume was set at 20 ​μL to ensure the accuracy and reliability of the analysis.

#### 2.2.3 Measurement of the Poly-Dispersity index (PDI), particle size (PS), and zeta potential (ZP)

The Zetasizer Nano-ZS Zen3600 by Malvern (Pennsylvania, USA) was used to assess the PDI, PS, and ZP values. The equipment was used to conduct all three parameter measurements, with triplicate measurements taken at a constant temperature of 25 ​± ​0.1​°C to guarantee precision. The samples were diluted 100 times with distilled water before analysis. [30].

#### 2.2.4 Entrapment efficiency

To determine the entrapment efficiency (EE%) of the nanoparticles, a precise procedure was meticulously followed. A 1 ​mL volume of either the cubosomal or SAIB-based nanodispersions was introduced into 10 ​kDa Amicon® ultrafiltration vials and then centrifuged for 15 ​min at 3000 ​rpm using PLC-012, Gemmy Industrial Corp., Taiwan. Following the centrifugation process, the drug concentration in the supernatant was analyzed using HPLC, with each preparation undergoing three cycles. The EE% was then calculated using (equation. 1), as outlined by Marathe et al., 2022 [[30], [31], [32]].

(1) $$\text{EE}\%=\frac{\text{The}\;\text{total}\;\text{amount}\;\text{of}\;\text{RXB}-\text{Amount}\;\text{of}\;\text{RXB}\;\text{in}\;\text{the}\;\text{supernatant}}{\text{The}\;\text{total}\;\text{amount}\;\text{of}\;\text{RXB}}\times 100$$

#### 2.2.5 Selecting the optimal formulas for cubosomal and SAIB-based nanodispersions

When choosing the best formulas for cubosomal and SAIB-based nano-vesicular (SBN) dispersions, there are several factors to consider. These factors include stability, size, and drug loading capacity, which can be determined by measuring particle size, PDI, PSD, and ZP. The One-way ANOVA test was used to identify significant differences among these variables. The ideal formulas were selected based on meeting the most conditions, such as having the smallest particle size, the lowest DPI, the highest ZP value (whether positive or negative), and the highest EE and yield. Any differences that were not statistically significant were disregarded.

#### 2.2.6 Lyophilization

In this study, Mannitol (MAN) was added to optimized cubosomal and SAIB-based nanodispersions in concentrations of 2.5%, 5%, and 10% w/v. The mixtures were vortexed for 5 ​min to ensure complete MAN dissolution, then frozen at −86°C in 50 ​mL Falcon tubes. After freezing, the dispersions were lyophilized for 24 ​h in a Christ Alpha 1-2LD plus (Munich, Germany) at −55°C and 7.6 ​kPa, following the protocol outlined in previous studies by ElMeshad et al., 2020 and Sheta et al., 2022 [33,34]. The lyophilized formulas were stored at room temperature for further investigation.

#### 2.2.7 Formulation of RXB tablets

The RXB tablets were made using a precise method. Firstly, a mixture of RXB-lyophilized formula containing 20 ​mg of RXB, Ac-Di-Sol® (Croscarmellose Sodium) (50 ​mg), and Mg-stearate (5 ​mg) was sifted through a mesh with a size of No.16 (1.18 ​mm) and then mixed. This mixture was then compressed using a Shimadzu IR hydraulic press at 6 ​kN (Kyoto, Japan) while monitoring the hardness of each tablet with the PharmaTest Tablet Hardness Tester (Hainburg, Germany). The tablet punch diameter was 11 ​mm round, with a beveled shape. The final product is a tablet with precise weight and hardness.

#### 2.2.8 Fourier-transform infrared spectroscopy (FTIR)

Using an IRAffinity-1S spectrometer (Kyoto, Japan), powder samples were analyzed using the LabSolutions Software. The spectral data were collected by scanning the samples within the range of 4000 to 500 ​cm^−1^ ​at room temperature.

#### 2.2.9 Differential scanning calorimetry (DSC)

The DSC analysis of the powder samples was performed using the Mettler Toledo DSC-1 instrument (Ohio, U.S.). To ensure accuracy, 5 ​mg of the sample was meticulously weighed into a perforated 40 ​μL aluminum pan and heated at 10​°C/min with a nitrogen gas flow of 50 ​mL/min. The Standard was an empty 40 ​μL aluminum. The STARe Software v12.10 developed by Mettler Toledo (Ohio, U.S) was utilized to analyze the data obtained from the experiment.

#### 2.2.10 Powder X-ray diffraction

The PXRD analysis was conducted with the EMPYREAN XRD analyzer (Almelo, Netherlands) under the National Research Center, Cairo, Egypt operation. The instrument utilized Cu Kα radiation (Y ​= ​1.54060) at 45 ​kV and 30 ​mA. The analysis was conducted by observing the pattern at a 4 ​°C/min rate and angles (°2Th) ranging from 10 to 50° [35].

#### 2.2.11 Evaluation of RXB tablets

##### 2.2.11.1 Tablet properties

Following the USP standards, both RXB and Xalerto tablets underwent weight variation, hardness, and friability tests. In weight variation test, 10 tablets of each were analyzed using RADWAG Analytical Balance, WL-110-0001 (Wroclaw, Poland), and the mean and standard deviation were calculated. The hardness was assessed by PharmaTest Tablet Hardness Tester, PTB111EP (Hainburg, Germany) [36]. Similarly, the friability test was conducted using PharmaTest single drum friability tester, PTF 10E (Hainburg, Germany). For this, 10 tablets were weighed, placed into the friability tester drum, and rotated for 4 ​min. Then, the tablets were cleaned of any dust, weighed again, and the percentage of weight loss was calculated [37].

##### 2.2.11.2 Disintegration time

The disintegration time was determined utilizing the PharmaTest Single Basket Tablet Disintegration Tester, PTZ-S (Hainburg, Germany). To achieve this, 800 ​mL of distilled water was heated to 37°C and served as the disintegration medium. Placing the tablets in the disintegrator basket, the time required for complete disintegration was recorded and measured as the disintegration time [38,39].

##### 2.2.11.3 Content uniformity

In order to gauge the concentration of RXB, the prepared RXB tablet and Xalerto tablet were mixed with 20 ​mL of dimethyl sulfoxide, stirred for 3 ​h, and subjected to sonication with a probe sonicator for 15 ​min. After filtration, the solution was diluted 100-fold in a chloroform: methanol solution (12.5:87.5, v/v%). Finally, the concentration of RXB was determined using HPLC, following the protocol by Lee et al., in 2021 [15].

##### 2.2.11.4 Dissolution testing

The optimized formula for cobosomal and SBN dispersion 20 ​mg RXB tablets (L4, L8) underwent dissolution testing and was compared to Xalerto's 20 ​mg tablets. The PharmaTest WS120S USP/EP Tablet Dissolution Tester (Hainburg, Germany) was used to conduct this test. The dissolution media comprised 900 ​ml of distilled water and was maintained at 37°C with a paddle speed of 100 ​rpm. At specific time intervals ranging from 5 to 60 ​min, 3 ​ml samples were taken, filtered through 0.45-μm filters, and diluted for HPLC RXB concentration measurement. The drug's release was calculated as cumulative percentages in each sampling interval to determine the dissolution profiles. The entire experiment was conducted in triplicate, following the methodology of Lee et al., 2021, and Meng et al., 2022 [15,21].

#### 2.2.12 Pharmacokinetic (PK) studies

A pharmacokinetic study was carried out on six male albino rabbits weighing between 2 and 3 ​kg, according to the guidelines and regulations of the Animal Ethics Committee of the Faculty of Pharmacy, Cairo University (No: PT2.1.4), and in full compliance with the ARRIVE guidelines 2020 [40]. The rabbits were housed in a controlled environment with a 12-h light and dark cycle and fed a pellet diet and water ad libitum. The study evaluated the in vivo performance of different formulations (XARELTO, L4, and L8).

After an overnight fast, the rabbits were given a dose of 1.55 ​mg/kg of each formulation and were fed again 4 ​h after dosing. The formulations were administered orally in 50 ​ml of water, with a washout period of 7 days between dosing. Blood samples were taken from the marginal ear vein at intervals ranging from 0.25 to 24 ​h after dosing and subsequently centrifuged and stored at −80​°C until analysis [8].

On the day of HPLC analysis, the plasma samples were defrosted at room temperature and prepared by mixing 100 ​μl aliquots of plasma with 100 ​μl of diphenhydramine internal standard (at a concentration of 500 ​ng/ml in acetonitrile) and 300 ​μl of acetonitrile. The resulting mixture was centrifuged and filtered through a 0.45 ​μm membrane filter before being analyzed using the HPLC method.

#### 2.2.13 Statistical analysis

One way ANOVA test was used to determine whether there are any significant differences between the means of three or more independent groups. Spearman's correlation, on the other hand, was used to measures the strength and direction of the relationship between two variables. Both of these tests were conducted using the GraphPad Prism 8.0.1. The results were displayed as mean and standard deviation. (P ​< ​0.05) was considered statistically significant [41].

## 3 Results and discussion

### 3.1 Physicochemical characterization of cubosomal and SAIB-based nanodispersions

Table 1 summarizes our observations on the physicochemical properties of cubosomal and SAIB-based nanodispersions. We discovered intriguing correlations between PS, PDI, ZP, EE, and yield. Specifically, our study revealed that increasing GMO concentration has an inverse relationship with PS, PDI, and ZP (p ​= ​0.0015; 0.0001; and <0.0001), indicating that higher GMO concentration leads to lower values for these variables. Conversely, increasing GMO content positively correlates with EE and yield (p ​= ​0.0092; 0.0249), suggesting that higher GMO content leads to higher EE and yield. Maintaining an optimal GMO concentration is crucial to ensure high-quality and consistent nanoparticle formulations, as PDI values depend heavily on GMO concentration [42,43]. Wei et al. (2021) also reported that GMO concentration significantly impacts ZP values, with increasing concentrations resulting in more negative ZP values [44]. Additionally, GMO concentration plays an important role in the formation of nanoparticles, acting as a stabilizer and emulsifier to prevent aggregation and sticking together during preparation. As GMO concentration rises, so do the values for EE and yield [45]. In addition, our study discovered that SAIB concentration has a negative correlation with PS and PDI (p = <0.0001; 0.0045), while EE and yield have a positive correlation with SAIB concentration (p ​= ​0.0029; 0.016). Thus, increasing SAIB concentration results in lower PS and PDI values but higher EE and yield.

### 3.2 Selecting the optimal formulas for cubosomal and SAIB-based nanodispersions

The ANOVA test was conducted)Table 2, Table 3 to analyze the significant differences between PS, PDI, ZP, EE, and Yield for both cubosomal and SAIB-based nanodispersions. The results revealed that in cubosomal dispersions, all parameters showed significant differences among the three preparations, except for EE%. Consequently, the preparation with the smallest PS, PDI, and ZP and the highest yield (F3) was selected. In SAIB-based nanodispersions, only PS and PDI showed significant differences, while ZP, EE, and yield did not exhibit any significant differences. Therefore, the preparation with the smallest particle size and PDI (F6) was chosen.

**Table 2:** The outcome of the ANOVA test for cubosomal dispersions.

	cubosomal dispersions			One Way ANOVA test	
	**F1**	**F2**	**F3**	**P value**	**P value summary**
PS (nm)	139.40 (±9.45)	130.00 (±7.54)	111.76 (±6.00)	0.013	∗
PDI	0.372 (±0.33)	0.273 (±0.46)	0.190 (±0.02)	0.002	∗∗
ZP (mv)	−11.76 (±0.75)	−17.96 (±1.30)	−24.99 (±1.28)	<0.0001	∗∗∗∗
EE (%)	68.53 (±3.46)	72.16 (±3.92)	80.36 (±6.81)	0.0642	ns
Yield (% w/w)	101.2 (±0.44)	99.67 (±0.29)	104.66 (±0.85)	<0.0001	∗∗∗∗

**Table 3:** The outcome of the ANOVA test for SAIB-based nanodispersions.

	SAIB-based nano-vesicular (SBN) dispersions			One Way ANOVA test	
	**F4**	**F5**	**F6**	**P value**	**P value summary**
PS (nm)	512.33 (±27.06)	252.53 (±3.93)	175.60 (±9.45)	0.0309	∗
PDI	0.737 (±0.04)	0.193 (±0.02)	0.254 (±0.05)	0.0074	∗∗
ZP (mv)	−3.58 (±0.46)	−3.73 (±0.56)	−4.03 (±0.67)	0.9667	ns
EE (%)	55.20 (±4.43)	54.93 (±4.52)	79.73 (±3.05)	0.4515	ns
Yield (% w/w)	100.07 (±0.25)	99.67 (±0.29)	101.5 (±0.36)	0.9985	ns

### 3.3 Selecting the optimal lyophilized cubosomal and SAIB-based nanodispersions

During our research, we utilized an ANOVA test to analyze the distinctions between PS, PDI, ZP, EE, and Yield for lyophilized cubosomal and SAIB-based nanodispersions. Our findings revealed that MAN concentration did not impact the properties of either lyophilized dispersion, as exhibited in Table 4, Table 5. Nevertheless, it is widely recognized that the concentration of MAN can influence the dissolution of poorly soluble drugs. For instance, a concentration of about 10–20% is commonly employed to increase drug solubility and dissolution rate [46]. However, the optimal concentration can vary based on the specific drug and formulation utilized. Consequently, we selected preparations with the highest MAN concentration (L4 and L8) based on the above findings.

**Table 4:** The outcome of the ANOVA test for lyophilized cubosomal formulas.

MAN (%w/v)	lyophilized cubosomal formulea				One Way ANOVA test	
	**L1**	**L2**	**L3**	**L4**	**P value**	**P value summary**
	**0**	**2.5**	**5**	**10**		
PS (nm)	111.77 (±6.00)	109.25 (±11.80	111.07 (±5.06)	105.43 (±10.97)	0.999	ns
PDI	0.19 (±0.02)	0.18 (±0.01)	0.18 (±0.01)	0.16 (±0.02)	0.9748	ns
ZP (mv)	−24.99 (±1.28)	−25.44 (±0.46)	−23.60 (±0.89)	−25.32 (±2.04)	0.9971	ns
EE (%)	80.37 (±6.81)	81.63 (±4.46)	83.99 (±3.33)	89.97 (±3.12)	0.9894	ns
Yield (% w/w)	101.20 (±0.44)	100.79 (±1.75)	101.00 (±0.84)	101.06 (±0.37)	>0.9999	ns

**Table 5:** The outcome of the ANOVA test for lyophilized SBN formulas.

MAN (%w/v)	lyophilized SBN formulea				One Way ANOVA test	
	**L5**	**L6**	**L7**	**L8**	**P value**	**P value summary**
	**0**	**2.5**	**5**	**10**		
PS (nm)	175.6 (±9.43)	156.4 (±10.9)	143.6 (±5.27)	113.8 (±12.4)	0.6647	ns
PDI	0.254 (±0.05)	0.223 (±0.03)	0.202 (±0.01)	0.171 (±0.02)	0.6076	ns
ZP (mv)	−4.033 (±0.67)	−4.467 (±0.74)	−4.250 (±0.68)	−5.817 (±1.29)	0.8541	ns
EE (%)	79.73 (±3.05)	81.74 (±3.65)	83.74 (±4.52)	88.56 (±5.20)	0.9887	ns
Yield (% w/w)	101.5 (±0.36)	99.00 (±3.31)	99.95 (±1.03)	101.99 (±3.22)	0.9998	ns

### 3.4 Fourier-transform infrared spectroscopy (FTIR)

**Fig. 2:**
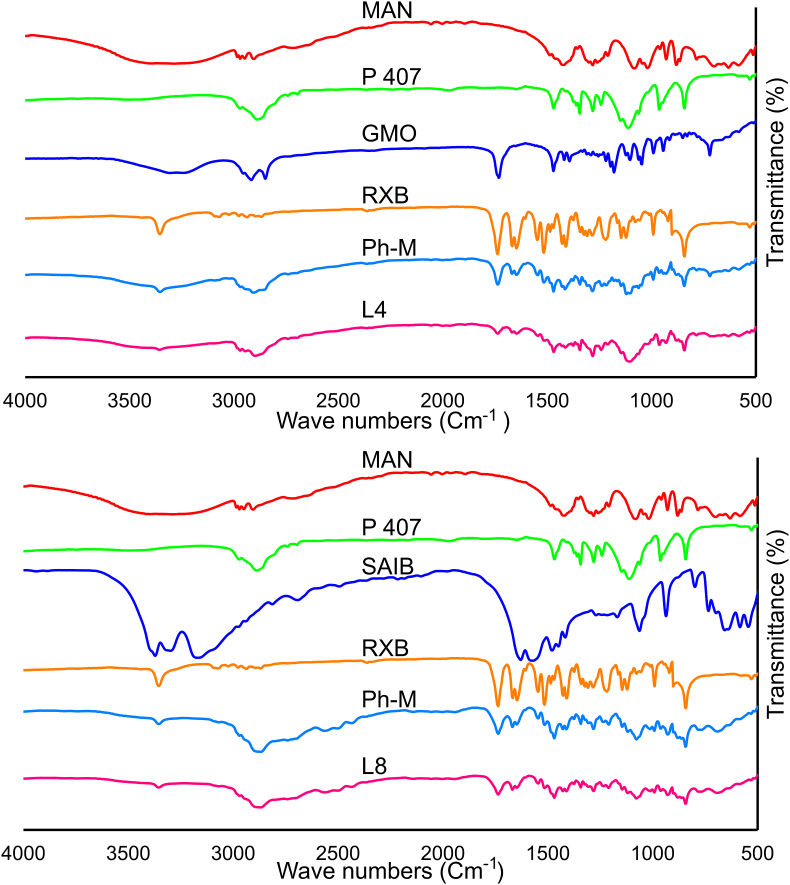
"FTIR Analysis of: Mannitol (MAN), Poloxamer 407 (P407) Glyceryle monooleate (GMO), Sucrose Acetate Isobutyrate (SAIB), Rivaroxaban (RXB), Physical mixtureI (Ph-M), The best lyophylaized cubosome (L4), and The best lyophylaized SAIB-based nanodispersions (L8)".

We have conducted FTIR on RXB, GMO, SAIB, P407, Physical mixture, L4, and L8, and obtained detailed results that provide insight into the chemical composition of these substances (Fig. 2). The FTIR curves for RXB indicate the presence of amine, carbonyl, and aromatic groups [47]. GMO shows characteristic peaks of C–H stretching and C=O bending [48]. SAIB displays absorption bands for C–O stretching and C=O stretching [49], while P 407 shows peaks of C–H stretching and C–O–C bending [50]. MAN is identified by its characteristic peaks, including O–H stretching, C–H stretching, and C–O bending, indicative of its chemical structure [51]. Finally, the FTIR results for the Physical mixture, L4, and L8 demonstrate the presence of all functional groups and characteristic peaks of individual components. Numerically, the FTIR spectra reveal that RXB has a peak at 1680 cm^-1^, GMO at 1700 cm^-1^, SAIB at 1740 cm^-1^, P 407 ​at 1100 cm^-1^, and MAN at 3300 cm^-1^, 2900 cm^-1^, and 1400 cm^-1^.

### 3.5 Differential scanning calorimetry (DSC)

**Fig. 3:**
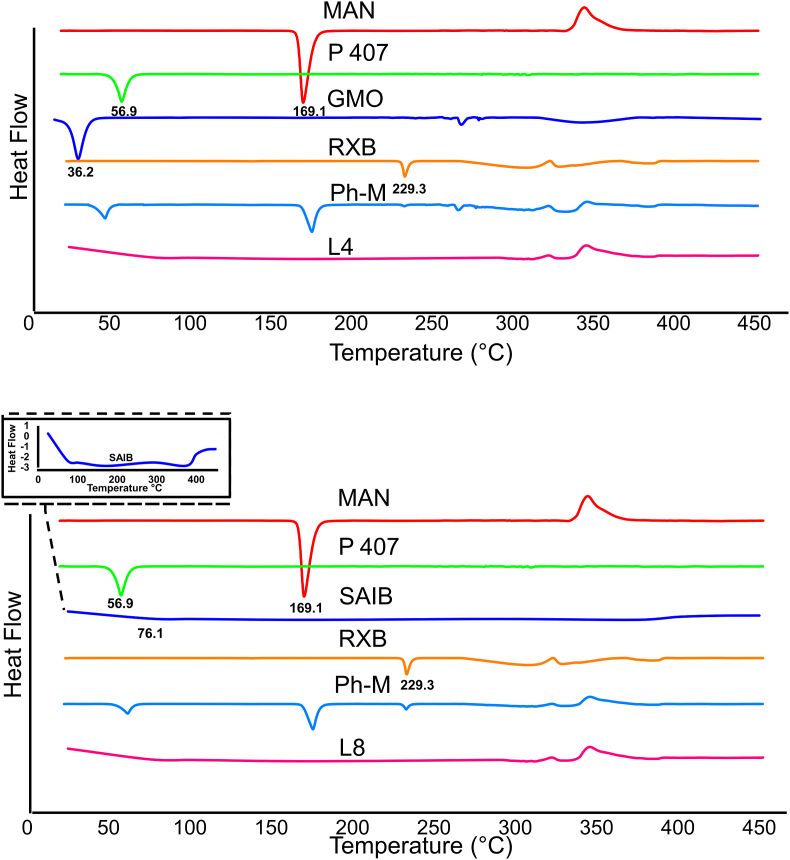
"Differential Scanning Calorimetry Analysis of: Mannitol (MAN), Poloxamer 407 (P407) Glyceryle monooleate (GMO), Sucrose Acetate Isobutyrate (SAIB), Rivaroxaban (RXB), Physical mixtureI (Ph-M), The best lyophylaized cubosome (L4), and The best lyophylaized SAIB-based nanodispersions (L8)".

In our analysis utilizing DSC (Fig. 3), we have found the following melting points and enthalpy values for each sample: RXB - melting point of 229.3°C and enthalpy of 259.1 ​J/g; GMO - melting point of 36.2°C and enthalpy of −31.9 ​J/g; SAIB - melting point of 76.1°C and enthalpy of 79.5 ​J/g; P 407 - melting point of −56.9°C and enthalpy of −52.6 ​J/g; and MAN - melting point of −169.1°C and enthalpy of −223.6 ​J/g. Our findings are consistent with previous studies that have reported similar results [[52], [53], [54], [55]]. Upon analyzing the physical mixture, we observed a broad endothermic peak on the DSC curve. This indicates that the mixture has a lower melting point and a higher enthalpy value than the individual substances. We measured the melting point of the physical mixture to be 50.5°C, and the enthalpy value was 243.9 ​J/g. The DSC curves of the physical mixture showed two endothermic peaks at 168.5°C and 228.7°C, indicating the melting points of MAN and RXB. We also examined the DSC curves of the selected formulas (L4 and L8). The curves displayed a broad endothermic peak at 60°C, indicating the presence of a mixture of solid and liquid phases.

### 3.6 Powder X-ray diffraction

**Fig. 4:**
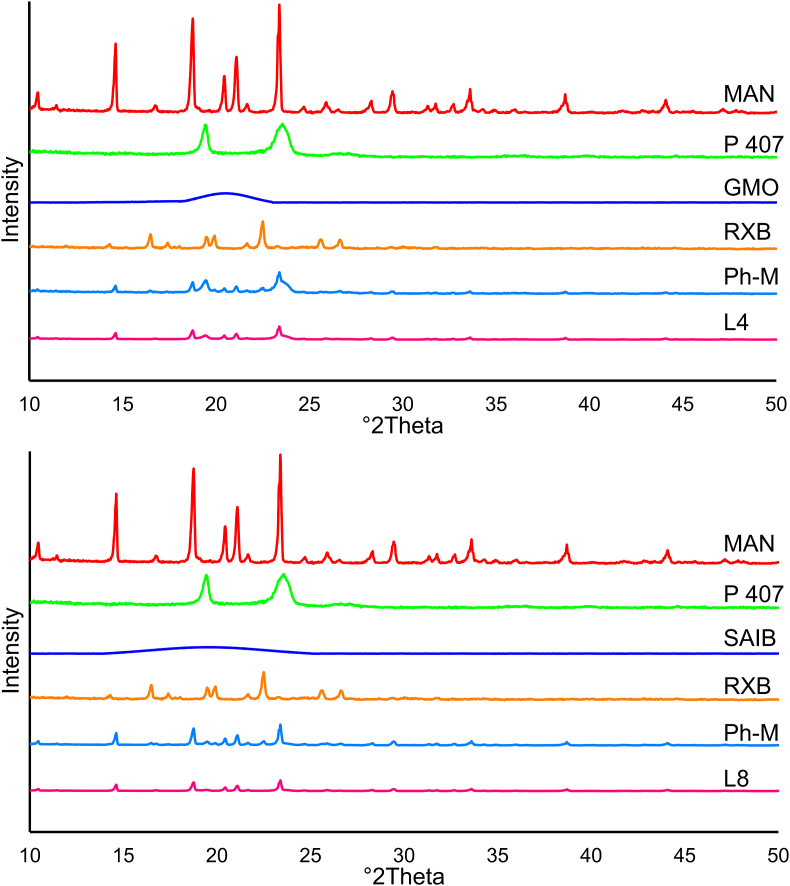
"Crystal Structure Analysis, Using Powder X-ray Diffraction-of: Mannitol (MAN), Poloxamer 407 (P407) Glyceryle monooleate (GMO), Sucrose Acetate Isobutyrate (SAIB), Rivaroxaban (RXB), Physical mixtureI (Ph-M), The best lyophylaized cubosome (L4), and The best lyophylaized SAIB-based nanodispersions (L8)" "

The results of the Powder X-ray diffraction test are quite fascinating. Based on the findings, RXB, GMO, and MAN show crystalline structures. On the other hand, SAIB and Polo 407 have an amorphous structure. Remarkably, the physical mixture sample combines crystalline and amorphous structures. Furthermore, Fig. 4 illustrate that L4 has an amorphous structure, while L8 has a highly amorphous structure.

### 3.7 Evaluation of RXB tablets

We have analyzed the weight variation, hardness, friability, disintegration time, and content uniformity of Xalerto tablets, L4 tablets, and L8 tablets, all containing 20 ​mg of RXB. Our findings revealed that the Xalerto tablets displayed a weight variation of (1.59% ​± ​0.18), the L4 tablets had a weight variation of (1.2% ​± ​0.31), and the L8 tablets had a weight variation of (1.3% ​± ​0.22). These results indicate that all preparations and Xalerto tablets meet the acceptable weight variation limit of 2%. When it comes to tablet hardness, the Xalerto tablets had a hardness of (8.5 kp ±1.22), the L4 tablets had a hardness of (9.2 kp ±1.43), and the L8 tablets had a hardness of (7.8 kp ±1.51), all of which fall within the acceptable range. Regarding friability, the Xalerto tablets had a friability of 0.5%, the L4 tablets had a friability of 0.4%, and the L8 tablets had a friability of 0.6%, all showing excellent resistance to friability. Moving on to disintegration time, the Xalerto tablets took 16 ​min to disintegrate, the L4 tablets took 10 ​min, and the L8 tablets took 12 ​min. It is important to note that the L4 tablets had the fastest disintegration time. Lastly, the Xalerto tablets had a content uniformity of (98.5% ​± ​2.05), the L4 tablets had a content uniformity of (99.2% ​± ​3.86), and the L8 tablets had a content uniformity of (98.8% ​± ​4.65), all of which meet the acceptable limit of content uniformity. Our tests indicate that the L4 tablets containing 20 ​mg of RXB are the ideal preparation, with the fastest disintegration time, highest hardness, lowest friability, and highest content uniformity. However, all three preparations meet the acceptable standards for weight variation, hardness, friability, disintegration time, and content uniformity.

### 3.8 Dissolution testing

**Fig. 5:**
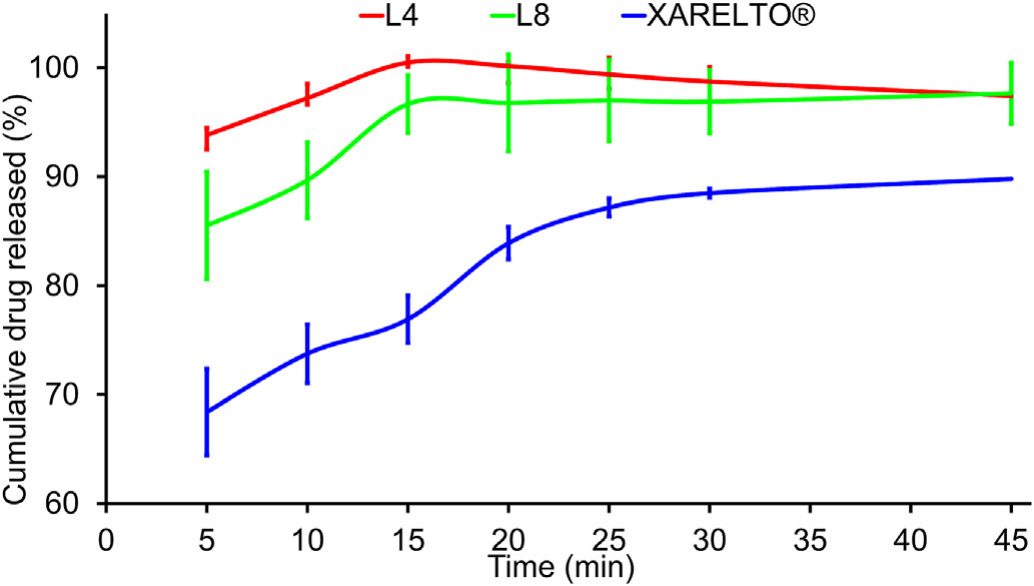
"In vitro dissolution profile of: The best lyophylaized cubosome (L4), The best lyophylaized SAIB-based nanodispersions (L8), and XARELTO®"

During the dissolution test (Fig. 5), drug release rates were measured at specific time intervals for each preparation. Results showed that L4 and L8 significantly improved the drug release rate compared to XARELTO® (p ​< ​0.05). This may be attributed to the enhanced solubility of RXB when encapsulated in nanosized particles, which have a larger surface area and promote better interaction with dissolution media. Furthermore, the small size of the particles reduces intermolecular forces between drug molecules. Among the three preparations, L4 demonstrated the best dissolution rate with a steady and consistent drug release rate throughout the test. While L8 had a slightly lower drug release rate, it still exhibited a relatively good dissolution rate.

### 3.9 Pharmacokinetic (PK) studies

**Fig. 6:**
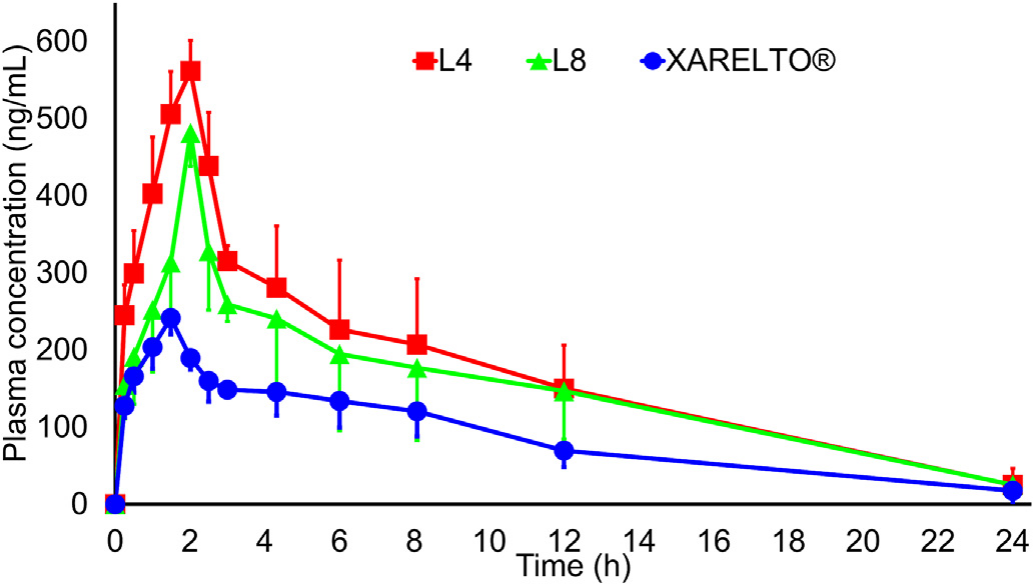
"Rabit Plasma concentration vs. time profile of: The best lyophylaized cubosome (L4), The best lyophylaized SAIB-based nanodispersions (L8), and XARELTO®"

In a pharmacokinetic (PK) study, the impact of administering 1.55 ​mg/kg of RXB was evaluated. To give the dosage, XARELTO®, L4, or L8 tablets were crushed, the exact amount was weighed, and then the powder was mixed with 50 ​ml of water. This was done to make it easier to administer to the rabbits, and they were given the suspension orally. The study results, illustrated in Fig. 6, show the plasma concentration/time profile and corresponding pharmacokinetic parameters. The AUC values for L4 and L8 were 4227.5 ​ng ​h/mL (± 168.5) and 3611.3 ​ng ​h/mL (± 157.9), respectively, while XARELTO® had an AUC value of 2117.0 ​ng ​h/mL (± 78.3). The Cmax values for L4 and L8 were 561.6 ​ng/mL (± 39.55) and 481.38 ​ng/mL (± 43.5), respectively, while XARELTO® had a Cmax value of 241.35 ​ng/mL (± 21.0). The Tmax values for L4 and L8 were 2.01 ​h (± 0.21) and 2.14 ​h (± 0.33), respectively, while XARELTO® had a Tmax value of 1.48 ​h (± 0.19). The t_1/2_ values for L4 and L8 were 4.31 ​h (± 2.11) and 4.45 ​h (± 3.23), respectively, while XARELTO® had a t_1/2_ value of 8.07 (± 6.19). The ANOVA test showed a significant difference between the AUC and Cmax values of L4, L8, and XARELTO® (p ​< ​0.05), but there was no significant difference between the Tmax and t_1/2_ values of the three formulations. The study indicates that L4 and L8 have better bioavailability than XARELTO® due to their improved solubility and dissolution profile. Upon oral administration, L4 and L8 interact with GI fluid in the GI tract, leading to increased solubility. The small size of the particles enhances their solubility, which increases bioavailability and improves therapeutic outcomes. Preparing drugs in nano form is a promising approach to enhancing their effectiveness in treating various diseases.

## 4 Conclusion

In conclusion, incorporating SAIB and GMO is critical to enhancing the dissolution and bioavailability of RXB in nano preparations, as demonstrated by our research. These compounds can improve the solubility and absorption of RXB, leading to more effective and efficient drug delivery systems in the pharmaceutical industry. Further investigation is necessary to fully comprehend the benefits and drawbacks of these ingredients in drug formulation. Our study underscores the significance of SAIB and GMO in drug formulation and their potential to transform drug delivery. Moreover, this innovative approach elevates the therapeutic effectiveness of RXB while minimizing the required dosage to achieve the desired outcome. The findings of our study pave the way for developing more effective drug delivery systems for various other APIs. Additionally, our research highlights nanotechnology's potential to revolutionize pharmaceutics and improve patient outcomes. By reducing particle size, nanotechnology has revolutionized drug delivery by enhancing drug solubility, bioavailability, and efficacy, leading to better therapeutic outcomes through improved absorption, distribution, and metabolism within the body.

## Contributions

**Adam A. Al-Shoubki:** formal analysis, methodology, validation, writing—original draft. **Mahmoud H. Teaima:** investigation, supervision, writing—review and editing. **Rehab Abdelmonem:** validation, supervision, writing—review and editing. **Mohamed A. El-Nabarawi:** conceptualization, investigation, visualization, supervision, study administration. **Sammar Fathy Elhabal:** investigation, writing—review and editing. All authors have read and approved the final manuscript.

## Funding information

This research received no funding.

## Ethics approval

All procedures of animal study were approved and regularly controlled by the Animal Ethics Committee of Faculty of Pharmacy Cairo University (No. PT2.1.4) and all experiments were performed in accordance with the guidelines and regulations of this committee. All the procedures were also carried out in full accordance with the ARRIVE guidelines 2020 [40].

## Declaration of competing interest

We, the authors of this manuscript, declare that there is no conflict of interest between us. We have no financial or personal relationships that could inappropriately influence our work. We have given full disclosure of any potential conflicts of interest to the Journal of Pharmaceutical Science Advances. We are committed to ensuring the integrity and objectivity of our research and writing, and we stand behind the accuracy and validity of our findings.
